# Depression and Anxiety Screening of Patients With Type II Diabetes Mellitus Attending Primary Healthcare Centers in Saudi Arabia

**DOI:** 10.7759/cureus.69393

**Published:** 2024-09-14

**Authors:** Morooj N Aldebani, Ahmad Y Saigh, Abdullah M Aljifri, Nahla Hariri

**Affiliations:** 1 Preventive Medicine, Saudi Board Program of Preventive Medicine, Makkah, SAU; 2 General Practice and Family Medicine, Sharae 7 Schemes Primary Healthcare Center, Makkah, SAU; 3 Internal Medicine, Saudi Board of Internal Medicine, Makkah, SAU; 4 Department of Community Medicine and Healthcare of Pilgrims, Umm Al-Qura University, Makkah, SAU

**Keywords:** diabetes complications, integrated care, makkah, mental health, quality of life

## Abstract

Background: Diabetes mellitus is a chronic condition affecting both physical and mental health. High blood sugar levels can lead to serious complications if not managed properly. Individuals with diabetes are also at a higher risk of developing mental health disorders like depression and anxiety.

Objective: The objective was to assess the prevalence of depression and anxiety among patients with type II diabetes attending primary healthcare centers in Makkah, Saudi Arabia.

Methods: An analytical cross-sectional study design was employed, targeting adults with type II diabetes attending primary healthcare centers in Makkah. A total sample size of 412 participants was selected through systematic random sampling. Data were collected using validated Arabic versions of the patient health questionnaire (PHQ-9) and the general anxiety disorder (GAD-7) questionnaire, along with demographic information. Statistical analysis was conducted with non-parametric tests used to evaluate the significance of outcome variables.

Results: The median age of participants was 47 years, with 237 (57.5%) being male and 373 (90.5%) Saudi nationals. Neuropathy was the most prevalent complication, affecting 181 (43.9%) of participants. The median GAD-7 score was 5, and the median PHQ-9 score was 7. Mild to moderate depression was observed in 224 (54.4%), and 194 (47%) had mild-to-moderate anxiety. Significant associations were found between mental health outcomes and several factors: nationality (non-Saudis showed higher depression and anxiety scores, p = 0.010 and p = 0.020, respectively), marital status (divorced participants had the highest mean ranks for both conditions, p = 0.003 for depression and p < 0.001 for anxiety), and monthly income (p = 0.007 for depression and p = 0.008 for anxiety). Additionally, the presence of diabetes complications was strongly associated with higher depression and anxiety scores (p < 0.001 for both).

Conclusion: The findings indicate a high prevalence of depression and anxiety among patients with type II diabetes in Makkah. These mental health issues are significantly associated with socio-demographic factors and diabetes complications. Integrated medical and psychological care is needed to improve the quality of life and treatment outcomes for diabetic patients.

## Introduction

Diabetes mellitus is a serious, chronic disorder characterized by hyperglycemia that develops due to the inability of the pancreas to produce a sufficient amount of insulin or the failure of the body to effectively utilize the produced insulin [1,2]. Diabetes may manifest with symptoms of thirst, frequent urination, blurred vision, unintentional weight loss, and fatigue. Diabetes is classified into type 1, type 2, gestational, and other specific types [3]. If untreated, diabetes can result in serious cardiovascular, ophthalmological, nephrological, neurological, and other complications [1].

According to the International Diabetes Federation (IDF), diabetes affects 537 million adults aged 20-79 years worldwide (prevalence, 10.5% of adults). The increase is alarming, especially in the Middle East and North Africa [2]. Diabetes prevalence estimates have reached 22% in Saudi Arabia in 2021 [4]. Diabetes has a significant impact both on human life and health expenditures [5]. The costs associated with diabetes care are 3.2 times greater than the mean per capita healthcare expenditure. This number rises to 9.4 times with diabetic complications [6].

Diabetes can lead to emotional and behavioral problems, which is not unusual considering the diagnosis is made for the entire lifetime, the potential debilitating complications, and the need to take complete responsibility for the disease management [7]. There is clear evidence demonstrating an association between diabetes and depression and/or anxiety [8,9]. In turn, these conditions may lead to poor treatment outcomes for diabetes, reduced quality of life, and increased healthcare costs [10].

Depression can cause feelings of sadness or a loss of interest in activities a person once enjoyed for a prolonged period [11]. Anxiety is an emotion described as feelings of tension, worried thoughts, and physical changes like increased blood pressure, sweating, tachycardia, etc. [8]. The prevalence of depression and anxiety was 279.6 and 301.39 million, respectively, in 2019, constituting 5% and 4% of the global population, respectively [12]. The prevalence of depression is two to three times higher among patients with diabetes than in the general population [13]. Some common determinants of depression and anxiety disorders among diabetes patients are female gender, family history or mental illness, a long history of diabetes (> five years), and the presence of retinopathy or kidney disease [13,14].

Type II diabetes mellitus (T2DM) is a chronic condition that significantly affects both the physical and mental health of individuals [9]. Its prevalence is particularly high in Saudi Arabia [4]. While the physical complications of diabetes are well-documented, there is increasing evidence of a strong association between diabetes and mental health disorders such as depression and anxiety [13]. These conditions can severely diminish the quality of life for diabetic patients, leading to poor treatment outcomes and higher healthcare costs. Given the high prevalence of diabetes in Saudi Arabia and the potential for concurrent mental health issues, understanding these associations is crucial. This study aims to assess the prevalence of depression and anxiety among patients with type II diabetes attending primary healthcare centers (PHCCs) in Makkah, Saudi Arabia.

## Materials and methods

Study design

This study utilized an analytical cross-sectional design to measure the prevalence of depression and anxiety among diabetic patients attending PHCCs in Makkah.

Study population

The study was conducted in Makkah, targeting adults with type II diabetes who were attending PHCCs in the area. Participants included in the study were adults (18 years and above) who had been diagnosed with diabetes and were currently attending PHCCs in Makkah. Participants with a history of severe mental illness or cognitive impairment, non-Arabic speaking individuals, and pre-diabetics were excluded from the study.

Sample size and sampling

The sample size was calculated using the equation for estimating a single proportion. Given a 50% prevalence (P = 0.5) and a desired margin of error of 5% (E = 0.05). The sample size (n) was calculated to be approximately 384 participants.

The study employed a comprehensive two-stage sampling technique to ensure a representative and unbiased sample from a total of 90 PHCCs in the Makkah region. In the first stage, a stratified random sampling method was used to select PHCCs across Makkah city. The city was divided into four geographical sectors (north, east, west, and south) by health authorities, and two PHCCs were randomly chosen from each sector, resulting in a total of eight PHCCs. In the second stage, a convenience sampling approach was utilized to recruit participants from these selected PHCCs. Eligible individuals visiting the PHCCs for any health-related reasons were approached and screened against the inclusion criteria. Those meeting the study requirements were invited to participate. 

Data collection

Data were collected using an electronic form designed on Google Forms (Google LLC, Mountain View, California, United States). The patient health questionnaire (PHQ-9) was used to identify the prevalence of depression, and the general anxiety disorder (GAD-7) questionnaire was used for anxiety screening. The Arabic versions of both questionnaires were tested and validated [15,16]. Participants were provided with clear instructions on how to complete the questionnaires, and their responses were automatically recorded in a secure database. In addition to the validated tools (PHQ-9 and GAD-7), demographic information such as age, gender, educational level, and duration of diabetes was collected to analyze the association between these factors and the prevalence of depression and anxiety among diabetic patients. The data collection process took place from March to July 2024, during weekdays from 8 AM to 4 PM. 

The scoring and cut-off values for the PHQ-9 were as follows: 0-4 indicated no depression, 5-9 indicated mild depression, 10-14 indicated moderate depression, 15-19 indicated moderate to severe depression, and 20-27 indicated severe depression. For the GAD-7, the scoring and cut-off values were: 0-4 indicated no anxiety, 5-9 indicated mild anxiety, 10-14 indicated moderate anxiety, and 15-21 indicated severe anxiety.

Statistical analysis

The data analysis was conducted using IBM SPSS Statistics for Macintosh, Version 29, (Released 2022; IBM Corp., Armonk, New York, United States). To assess the normality of continuous variables, the Kolmogorov-Smirnov test was employed, revealing a skewed distribution. Consequently, these variables were summarized using medians and interquartile ranges (IQRs). Frequencies and proportions were computed for categorical variables. Reliability was tested for the PHQ-9 and GAD-7 scales and showed high-reliability scores using Cronbach's alpha. The PHQ-9 reliability score was r=0.895, and the GAD-7 reliability score was r=0.896. The total scores for PHQ-9 and GAD-7 served as outcome variables, and their statistical significance was evaluated using the Mann-Whitney U test and the Kruskal-Wallis test. These non-parametric tests reported the mean rank and p-value, with higher mean ranks indicating elevated levels of the measured parameters. A significance threshold was set at p < 0.05.

Ethical considerations

This study received ethical approval from the Institutional Review Board (IRB) of the Ministry of Health at Makkah, Saudi Arabia (IRB No.: H-02-K-076-1223-1051). Data confidentiality and security were strictly maintained, ensuring that all personal information was anonymized. Only authorized research team members had access to the collected data. Additionally, the data were used exclusively for research purposes, and any findings presented or published were done so in an aggregated and anonymized manner to protect participants' privacy.

## Results

The total sample size for this study was 412 participants. The ages of the participants ranged from 20 years to 88 years, with a median age of 47 years (IQR: 37.5-56). In terms of gender distribution, 237 participants (57.5%) were male, and the majority of the participants were Saudi nationals, accounting for 373 individuals (90.5%). Regarding marital status, 60 participants (14.6%) were single, 271 (65.8%) were married, 40 (9.7%) were divorced, and 41 (10.0%) were widowed. The majority, 227 (55.1%), indicated having a bachelor's degree; 270 participants (65.5%) were employed, and 120 participants (29.1%) earned ≤ 5,000 Saudi riyals (SAR). In terms of body mass index (BMI), the vast majority, 390 participants (94.7%), were obese. The demographic characteristics are shown in Table 1.

**Table 1:** Demographic characteristics of the study participants SAR: Saudi riyals

Variable	Group	N	%
Gender	Male	237	57.5%
	Female	175	42.5%
Nationality	Saudi	373	90.5%
	Non-Saudi	39	9.5%
Marital status	Single	60	14.6%
	Married	271	65.8%
	Divorced	40	9.7%
	Widow	41	10.0%
Educational level	No official education	32	7.8%
	Less than high school	26	6.3%
	High school	52	12.6%
	Diploma	38	9.2%
	Bachelor	227	55.1%
	Higher education	37	9.0%
Job status	Employed	270	65.5%
	Unemployed	142	34.5%
Monthly income (SAR)	≤5,000	120	29.1%
	5,001-10,000	87	21.1%
	10,001 – 15,000	117	28.4%
	>15,000	88	21.4%
Body mass index	Underweight	16	3.9%
	Normal	3	0.7%
	Overweight	3	0.7%
	Obese	390	94.7%

**Figure 1:**
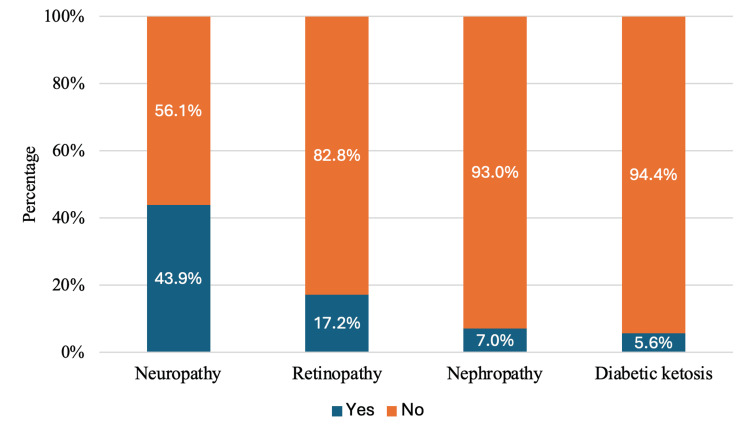
Distribution of diabetes complications among the study participants

Among the study participants, a total of 228 individuals (55.3%) reported being diagnosed with diabetes complications. Neuropathy was identified as the most prevalent complication, affecting 181 participants (43.9%). Retinopathy was the next most common, reported by 71 participants (17.2%). Nephropathy was present in 29 participants (7.0%), while diabetic ketosis was noted in 23 participants (5.6%). This distribution is demonstrated in Figure 1.

**Figure 2:**
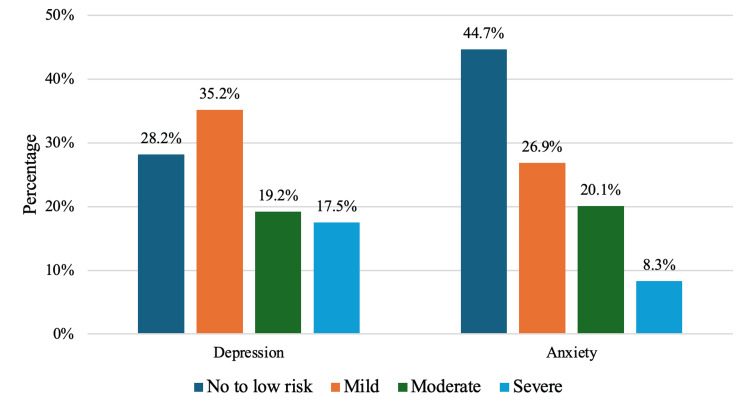
Distribution of depression and anxiety among diabetic patients

The GAD-7 score ranged from 0 to 21, with a median score of 5 (IQR: 2-10). The PHQ-9 total score ranged from 0 to 27, with a median score of 7 (IQR: 3-12). Notably, 224 participants, 54.4% exhibited mild-to-moderate depression, while 72 participants, 17.5% had severe depression. For anxiety, 194 participants, 47%, reported mild-to-moderate levels, and 34 participants, 8.3% experienced severe anxiety. These findings highlight the high prevalence of mental health issues in patients with type II diabetes. Further assessment was done of the impacts of depression and anxiety on daily functioning. For PHQ-9, 165 participants, 40% of the participants, experienced no difficulty managing depressive symptoms, 167 participants, 40.5%, found them somewhat difficult, 66 participants, 16%, found them very difficult, and 14 participants, 3.4%, found them extremely difficult. Similarly, the GAD-7 results showed that 153 participants, 37.1%, had no difficulty managing anxiety symptoms, 175 participants, 42.5%, found them somewhat difficult, 73 participants, 17.7%, found them very difficult, and 11 participants, 2.7%, found them extremely difficult. These findings highlight the need for comprehensive mental health support for patients with T2DM. Detailed severity distributions are shown in Figure 2. 

An in-depth analysis of the depression and anxiety scores among participants revealed several significant socio-demographic factors. Nationality emerged as a significant determinant, with non-Saudis exhibiting higher mean ranks for both depression (mean rank = 253.22, p = 0.010) and anxiety (mean rank = 248.67, p = 0.020) compared to Saudis. Divorced participants had the highest mean ranks for both depression (mean rank = 268.75, p = 0.003) and anxiety (mean rank = 294.38, p < 0.001). Monthly income significantly impacted both depression (p = 0.007) and anxiety (p = 0.008) scores, with participants earning between 5,001 and 10,000 SAR demonstrating the highest mean ranks. Additionally, despite the variation in the sample size of BMI groups, underweight had significantly higher anxiety scores (p = 0.006) compared to other BMI groups. The comparison of demographic characteristics is further detailed in Table 2.

**Table 2:** Depression and anxiety mean ranks by demographic characteristics with corresponding p-values SAR: Saudi riyals

Variable	Group	Depression mean rank	p-value	Anxiety mean rank	p-value
Gender	Male	197.84	0.085	200.93	0.268
	Female	218.23		214.05	
Nationality	Saudi	201.62	0.010	202.09	0.020
	Non-Saudi	253.22		248.67	
Marital status	Single	204.85	0.003	204.59	<0.001
	Married	202.06		201.69	
	Divorced	268.75		294.38	
	Widow	177.56		155.38	
Educational level	No official education	175.27	0.444	146.09	0.064
	Less than high school	201.12		187.25	
	High school	210.36		213.84	
	Diploma	214.58		214.67	
	Bachelor	213.25		211.51	
	Higher education	182.18		222.81	
Job status	Employed	204.45	0.630	212.37	0.166
	Unemployed	210.39		195.34	
Monthly income (SAR)	≤5,000	214.64	0.007	214.64	0.008
	5,001-10,000	236.79		236.79	
	10,001 – 15,000	180.62		180.62	
	>15,000	199.86		199.86	
Body mass index group	Underweight	150.47	0.229	107.66	0.006
	Normal	216		248.67	
	Overweight	155.67		162.5	
	Obese	209.12		210.57	

Participants diagnosed with diabetes complications had significantly higher depression and anxiety mean ranks compared to those without complications, with mean ranks of 239.3 (p < 0.001) and 233.95 (p < 0.001), respectively. Specifically, participants with neuropathy showed higher depression and anxiety scores (233.78 and 232.38, p < 0.001). Those with retinopathy also had higher scores (284.09 and 277.67, p < 0.001). Nephropathy was linked to elevated scores (287.22 and 287.84, p < 0.001). Diabetic ketosis participants reported higher scores (276.91 and 276.17, p = 0.003 and p = 0.004). The detailed results are presented in Table 3.

**Table 3:** Depression and anxiety mean ranks and corresponding p-values by diabetes complications among study participants

Complication	Response	Depression mean rank	p-value	Anxiety mean rank	p-value
Were you diagnosed with diabetes complications?	Yes	239.3	<0.001	233.95	<0.001
	No	165.86		172.48	
Neuropathy	Yes	233.78	<0.001	232.38	<0.001
	No	185.13		186.23	
Retinopathy	Yes	284.09	<0.001	277.67	<0.001
	No	190.34		191.68	
Nephropathy	Yes	287.22	<0.001	287.84	<0.001
	No	200.39		200.34	
Diabetic ketosis	Yes	276.91	0.003	276.17	0.004
	No	202.34		202.38	

## Discussion

The findings of our study further substantiate the significant burden of mental health disorders among patients with T2DM, particularly in the context of primary healthcare settings in Makkah. Our results indicate a pronounced prevalence of mild-to-moderate depression among 224 individuals (54.4%) and anxiety among 194 individuals (47%), with severe cases also notable. Socio-demographic factors such as nationality and marital status were significantly associated with mental health outcomes, with non-Saudis and divorced individuals showing higher mean ranks for depression and anxiety. These findings align with multiple studies conducted globally and within the region, underscoring the significant burden of mental health disorders among diabetic patients [17-20].

The prevalence rates of mild-to-moderate depression and mild-to-moderate anxiety in our study are consistent with the results from a systematic review conducted in South Asia, which reported a pooled prevalence of depression at 40% among diabetic patients [17]. Lower prevalence rates have been observed in other regions; for instance, a study in Ecuador reported depression and anxiety rates of 31.7% and 33.7%, respectively, among T2DM [18]. In the Middle East, a recent systematic review reported depression prevalence as high as 74.4% among T2DM, reflecting substantial variability but generally high rates in the region [19].

The significant association between socio-demographic factors and mental health outcomes in our study mirrors findings from other research. For example, divorced participants in our study exhibited the highest mean ranks for both depression and anxiety, a trend also observed in another study in the Netherlands [20]. Similarly, our finding that non-Saudis had higher depression and anxiety scores is consistent with the literature, indicating that minority status and socio-economic factors can influence mental health outcomes in diabetic patients [21]. The interplay between obesity and mental health in our cohort is also notable. Despite the majority of our sample being classified as obese (94.7%), the highest anxiety scores were observed among the underweight group, albeit a smaller subset. This paradox highlights the complex relationship between body weight and psychological health, where underweight individuals may face unique stressors, including malnutrition and frailty, contributing to heightened anxiety [22,23].

The impact of diabetes complications on mental health observed in our study is well-documented in the literature. Participants with neuropathy, retinopathy, and nephropathy showed higher depression and anxiety scores, corroborating findings from studies in Saudi Arabia and elsewhere that link these complications with poorer mental health [24]. Alshabanat et al. (2023) and Baghdadi et al. (2021) also highlighted the association between nephropathy and increased anxiety and depression among diabetic patients in Saudi Arabia [25,26]. Our results underscore the need for integrated medical and psychological care for diabetic patients, particularly those with complications. This necessity is echoed by Alosaimi et al. (2022), who reported that longer diabetes duration and multiple complications are significant risk factors for depression among diabetic patients [27]. While our study did not include the perceived trust in healthcare workers, AlRuthia et al. (2020) emphasized the role of trust in healthcare providers in mitigating anxiety and depression, suggesting that improving patient-provider relationships could be beneficial [28]. 

The study offers important insights into the prevalence and causes of depression and anxiety among T2DM patients in primary care centers in Makkah, but it has limitations. Self-reported data may introduce bias, the predominance of obese individuals limits generalizability, and potential confounding factors like lifestyle and other comorbidities were not included. Despite these issues, this study is among the few examining T2DM patients' mental health in Makkah, Saudi Arabia, filling a crucial gap. The large sample size adds robustness, and the use of validated instruments (GAD-7 and PHQ-9) ensures reliable findings. It also identifies key socio-demographic and clinical factors linked to mental health, offering insights for targeted interventions. Including a diverse sample in terms of nationality and marital status provides a nuanced understanding of mental health influences among T2DM patients.

## Conclusions

The study reveals that nearly half of the participants exhibit mild to moderate depression and anxiety, with a significant portion suffering from moderate to severe levels. Key findings indicate that socio-demographic factors such as nationality, marital status, and monthly income are significantly associated with depression and anxiety scores. Non-Saudis and divorced individuals are particularly prone to elevated levels of these mental health issues. Additionally, diabetes-related complications markedly increase the risk of depression and anxiety among patients. The strong association between mental health disorders and diabetes complications underscores the necessity for an integrated approach to diabetes management that includes both medical and psychological care. These findings highlight the critical need for comprehensive mental health screening and support programs within primary healthcare settings to improve overall health outcomes and quality of life for patients with T2DM. The study's findings advocate for the implementation of comprehensive mental health screening and support programs within primary health care settings. Such initiatives should focus on early identification and intervention for depression and anxiety to mitigate their adverse impacts on diabetes management and patient well-being.
